# Impact of Chemical and Biological Fungicides Applied to Grapevine on Grape Biofilm, Must, and Wine Microbial Diversity

**DOI:** 10.3389/fmicb.2018.00059

**Published:** 2018-02-02

**Authors:** Rocío Escribano-Viana, Isabel López-Alfaro, Rosa López, Pilar Santamaría, Ana R. Gutiérrez, Lucía González-Arenzana

**Affiliations:** Instituto de Ciencias de la Vid y del Vino, ICVV (Gobierno de La Rioja, Centro Superior de Investigaciones Científicas and Universidad de La Rioja), Logroño, Spain

**Keywords:** *Botrytis cinerea*, grape biofilm, must, wine, microbiota, biofungicide, diversity, species richness

## Abstract

This study was aimed to measure the impact of the application of a bio-fungicide against *Botrytis cinerea* on the microbiota involved in the alcoholic fermentation (AF) of Tempranillo Rioja wines. For this purpose, a bio-fungicide composed of the biological control bacterium *Bacillus subtilis* QST713 was applied to the vineyard. The microbial diversity was analyzed from grape biofilm to wine. Impact on microbial diversity was measured employing indexes assessed with the software PAST 3.10 P.D. Results were compared to non-treated samples and to samples treated with a chemical fungicide mainly composed by fenhexamid. Overall, the impact of the biological-fungicide (bio-fungicide) on the microbial diversity assessed for grape biofilm and for musts was not remarkable. Neither of the tested fungicides enhanced the growth of any species or acted against the development of any microbial groups. The bio-fungicide had no significant impact on the wine microbiota whereas the chemical fungicide caused a reduction of microbial community richness and diversity. Although environmental threats might generate a detriment of the microbial species richness, in this study the tested bio-fungicide did not modify the structure of the microbial community. Indeed, some of the *Bacillus* applied at the grape surface, were detected at the end of the AF showing its resilience to the harsh environment of the winemaking; in contrast, its impact on wine quality during aging is yet unknown.

## Introduction

Traditionally referred to as the “gray mold”, *Botrytis cinerea* is a necrotrophic pathogen able to rot grapes that negatively affects must and wine organoleptic quality (Cantoral et al., 2011).

Most grape growers have been fighting against this mould using chemical fungicides. Viticulture is one of the most pesticide consuming crops in spite of its low production rates, what is linked to the sudden resistance that some mould develop to chemical fungicides (Provost and Pedneault, 2016). This situation has encouraged the almost urgent seeking of alternative control tools (Garrido et al., 2017).

Chemical fungicides based on copper molecules have been reported as a drawback for the maintenance of the ecological balance of ecosystems. Consequently, some limits in their use on crops have been established (Provost and Pedneault, 2016). For this reason, copper free chemical fungicides, which are thought to be harmless to the environment, are currently being commercialized. On another note, consumers are demanding organic products manufactured without chemicals and preservatives (D’Amico et al., 2016). This trend has been extended to oenological industry, what has meant an advance in researching new bio-products to be employed in grapevines as a biocontrol strategy.

As a result, some industries have struggled to apply biological fungicides (bio-fungicides) to the grapevine. This type of commercial products has been inspired in the biological control carried out on other crops affected by similar diseases. The application in the vineyard of some yeasts such as *Candida sake* (Garrido et al., 2017) or of some bacteria such as *Bacillus subtilis* strains (Pertot et al., 2017) has been aimed to reduce some grapevine diseases. Some of these bio-fungicides mean a great advantage compared to chemical products because they can be applied in the grapevine from full bloom to only two or three days before being harvested. Indeed, this organic viticulture tries to reduce the employment of pesticides without altering the production and yields of grapevine.

Oenology is featured for being one of the few food industries in which the raw material and even the elaboration process are not under sterile conditions. For a start, the grapevine ecosystem is the first one involved in the winemaking. In effect, the grapes before the harvest held plenty of microorganisms belonging to yeast and bacteria microbial groups. These yeasts are usually S*accharomyces* and non-*Saccharomyces* genera whereas acetic acid bacteria (AAB), lactic acid bacteria (LAB), and environmental bacteria (EB) represent the bacterial group. Once grapes have been harvested and introduced in the winery, the microbial community established on the biofilm of the grape surface is blended with the own winery microbiota that persists from one vintage to the next one. When grapes are manufactured, usually including destemming and crushing, the microbial community strikes a balance and then the alcoholic fermentation (AF) begins. *Saccharomyces* yeasts that change the must into wine develop this fermentative stage. After AF, the malolactic fermentation could take place. This stage is based on the biological deacidification of malic acid into lactic acid and it is mainly carried out by the LAB *Oenococcus oeni*. Regarding the mentioned above, the winemaking could be one of the most complex microbiological transformation in the food industry.

Furthermore, recent published results have reported the presence of microorganisms during the whole winemaking that are not usually involved in oenological process. Some of these microorganisms have not been deeply analyzed in wines; for instance, some AAB that were thought to be in the early stages of winemaking have been detected in middle AF (Portillo et al., 2016) or some EB genera from open environments that have been also detected in must even after being sulphited (González-Arenzana et al., 2017a).

Overall, microbial ecology studies in wines are usually based on Polymerase Chain Reaction (PCR) of the DNA extracted from colonies (culture-dependent method) and the DNA extracted from Denaturing Gradient Gel Electrophoresis (DGGE) bands, followed by sequencing of amplicons for their later identification. Moreover, the combination of both approaches has been demonstrated to be interesting to tackle ecological studies (González-Arenzana et al., 2017a,b).

As far as it is concerned, this is the first study of the microbial diversity during the whole winemaking in relation to fungicide application in the grapevine. For this reason, this study was aimed to know if the ecological balance of the microbial communities of grape biofilms, musts and wines were altered by the application of a biological fungicide mainly composed by the *Bacillus subtilis* QST713. In order to achieve a general and real approach, results were compared with the impact of a chemical fungicide, based on fenhexamid molecule. Both fungicides were prescribed against the gray mold *Botrytis cinerea.*

## Materials and Methods

### Grapevine Treatments and Sampling

The project was carried out in a *Vitis vinifera* L. cv. Tempranillo vineyard located in the Rioja qualified Designation of Origin (D.O.C. Rioja). The vineyard was managed under conventional soil tillage with approximately 3530 plants per 100 square meters (Ha). An experimental design with randomly established blocks of four replicates per treatment was performed (12 replicates) in the vineyard. Replicates of the same sample were located in the same row; samples were contiguous and separated by an average distance of 2.7 m. Each replicate received the same agronomic management previously to the treatments. The vineyard had not symptoms of being affected by *Botrytis cinerea* at the beginning of the study.

Three treatments were performed in the same vineyard in order to avoid biases caused by the climatic or the agronomic conditions. Treatments were applied with an automatic knapsack sprayer. One was referred to as “C” (control) because no fungicide was applied. Other treatment was applied with a dose of 4 kg/Ha twice, 21 days and 3 days before harvest was referred to as “Bio”. This later was based on the application of a wet powder product that was a biological fungicide with 5.3 × 10^10^ colony forming units (CFU) per millilitre (mL) of the *Bacillus subtilis* strain QST 713 (Serenade^®^ Max, Bayer Crop Bioscience S.L.) The other treatment referred to as “Chem”, consisted of the application of a traditional chemical fungicide product based on fenhexamid chemical compound (Teldor^®^, Bayer Crop Bioscience S.L.) 21 days before harvest (1.7 kg/Ha). The average number of plants per replicate was 25. Control of ripening was performed from veraison stage to the optimal date for harvest. Each replicate was separately harvested and vinified.

Sampling was distributed in three moments. Firstly, the microbiota of grape surfaces was sampled in the vineyard one day before harvest. At this initial stage, 500 g of grapes of each replicate and treatment were randomly selected in the vineyard and incubated in 500 mL of sterile isotonic solution (PT: 0.1% soy peptone and 0.01% Tween 80) (Prakitchaiwattana et al., 2004) in an orbital shaking (80 rpm) for 2 h at room temperature. After shaking, the biomass was recovered by centrifugation (30 min, 10,000 × *g*, 4°C) and the pellet was then suspended in 15 mL of PT. Oenological parameters of the must such as probable alcohol, pH, and total acidity were analyzed according to ECC official methods (European Community, 1990). Moreover, gluconic acid was determined by an automated enzymatic method (Miura One, TDI, Spain).

The second sampling stage was performed in the experimental winery 48 h after filling tanks of 100 L with crushed, destemmed and sulphited grapes (50 mg/L SO_2_). So, that 15 mL of each must was sampled. The AF was spontaneous and the decreasing density was daily controlled (data no shown). When AF was completed, the third sampling took place and 15 mL of each replicate wine were sampled.

### Culture Dependent Identification by Ribosomal DNA Sequencing

The viable and cultivable (VC) microbial community of each replicate and treatment was analyzed. For this purpose, several dilutions of the initial samples of PT, must and wines were spread on different culture media plates. The VC yeast community was quantified employing two culture media, GYP for total yeast community incubated at 28°C during 48 h (González-Arenzana et al., 2017a) and DBDM for *Dekkera/Brettanomyces* detection incubated at 25°C for 2 weeks in anaerobic conditions (Gas Pak System, Oxoid Ltd., Basingstoke, England) (Rodrigues et al., 2001; Guzzon et al., 2011). The VC bacteria community was quantified employing two culture media; MRS (De Man et al., 1960) for total LAB, incubated at 28°C for 48 h in anaerobic conditions and Mann (González-Arenzana et al., 2017a), for AAB and EB, at 25°C for 48 h. As soon as had the incubation period finished, the cells growing (CFU/mL) on the different culture media were counted and expressed in logarithmic units (log).

Plates with VC microbial communities between 1 and 2 log units were reserved for randomly isolation of 10 colonies. Genera and species identification of VC microbial community was carried out by ribosomal DNA sequencing. In case of yeasts, partial 26S rRNA genes were amplified using the primers NL1 and NL4 (Cocolin, 2000); for LAB species identification, the PCR was performed with primer pairs WLAB1 and WLAB2 targeted the V4 and V5 16S rDNA regions as López et al. (2003) described. Finally, the AAB and the EB species identification was performed by amplification of the V1 to V6 region of 16S rDNA gene with 8F and 907R primers (Posada et al., 2016). Macrogen Inc. (Seoul, South Korea) sequenced the PCR amplicons. Then, sequences were compared to GenBank nucleotide database using the Basic Local Alignment Search Tool (BLAST) (Altschul et al., 1990). The identification was considered correct when gene sequences showed identities of at least 98%.

### Culture Independent Identification by PCR-DGGE

The biomass obtained from grapes in PT, from musts and from wines was frozen at –80°C in a volume of 10 mL. After this, the DNA was directly extracted from these samples following the protocol described by González-Arenzana et al. (2013).

The DNA extracted from samples was amplified by different PCRs that were run in an Applied Biosystem, GeneAmp^®^ PCR System 2700 thermocycler in a final volume of 50 μL with 2 μL of the extracted DNA (approximately 10 ng) as described González-Arenzana et al. (2017a). For yeasts and moulds, the D1 region of the 26S rRNA gene was amplified using the primers NL1^GC^ and LS2 (Cocolin, 2000). For bacteria, the V7 to V8 region of 16S rDNA gene was amplified with WBAC1 and WBAC2^GC^ primers and with WLAB1 and WLAB2^GC^ (López et al., 2003). The PCR reactions were performed following the indications González-Arenzana et al. (2013). An aliquot (5 μL) of the amplified DNA was analyzed by 1% agarose gel electrophoresis to verify that the PCR worked prior to DGGE.

The DGGE was carried out to separate the respective amplicons with the D-CODE^TM^ universal mutation detection system (Bio-Rad, Hercules, CA, United States). PCR products were run on 8% (wt/vol) polyacrylamide gels in a TAE buffer (2 M Tris, 1 M glacial acetic acid and 50 mM EDTA pH 8) at a constant temperature of 60°C. The urea-formamide content ranged from 35 to 60% for NL1^GC^-LS2 amplicons, and from 35 to 55% for WLAB1-WLAB2^GC^ amplicons and from 35 to 65% for WBAC1-WBAC2^GC^. An initial stage of electrophoresis was performed (10 min at 20 V) and after this, the electrophoresis products run for 18 h with a voltage of 80 V. Then, gels were stained in ethidium bromide and visualized with UV *trans*-illumination (GelDoc, Bio-Rad). Blocks of the polyacrylamide gels with the selected DGGE bands were excised and incubated overnight in 20 μL of sterile, pure water at 4°C to make DNA bands diffuse to the liquid. One microliter of this elution was re-amplified using the PCR conditions described above with primers without the GC clamp. Macrogen Inc. (Seoul, South Korea) purified and sequenced by the PCR amplicons. Sequences were compared to the GenBank nucleotide database with BLAST. The identification was considered correct when gene sequences showed identities of at least 98%.

### Measurement of Diversity and Structure Community and Statistical Analysis

Alpha diversity parameters were assessed by the software PAST 3.10 P.D. (Ryan et al., 1995) analyzing the detected species in each of the replicates (*n* = 4) of the three treatment. For each replicate, the average number of detected species (S) and the Margalef index that supposes a functional relation between the number of species and the total number of individuals (Margalef, 1958) were calculated for describing the richness of species of each sample. On another note, the structure of the studied microbial communities was determined by dominance indexes such as Simpson and Berger–Parker, and finally by Shannon–Wiener equity index and by the non-parametric index Chao1. The Simpson index measures the possibility that two randomly chosen individuals belong to the same species (Fedor and Spellerberg, 2013). Opposite in meaning, the Berger–Parker index measures the dominance in individuals of the dominant taxon (Harper, 1999). Finally, the entropy of the community was measured by the Shannon–Wiener index (*H*) that takes into account the number of individuals as well as the number of species (Karydis and Tsirtsis, 1996; Death, 2008). The Chao1 is an estimator of the species number based on the odd species (Portillo et al., 2016).

Data of counts and diversity indexes of each replicate (*n* = 4) were processed using the variance analysis (ANOVA) with the Tukey tests (at *p* ≤ 0.01) using the software IBM SPSS Statistic 20.0 (Chicago, United States). Hierarchical cluster with all the information of diversity indexes regarding each sample was constructed with the same software.

## Results

Initial oenological parameters of grapes from control, bio-fungicide, and chemical fungicide application were quite similar between samples. In effect, the must from control grapes had a probable alcohol degree (% v/v) of 13.5, a pH of 3.38 and a total acidity (g/L tartaric acid) of 6.45. Must from bio-fungicide application had a probable alcohol degree of 13.0% v/v, a pH 3.33, and a total acidity of 6.41 g/L. Eventually, must from chemical fungicide application had a probable alcohol degree of 13.2% v/v, a pH 3.37, and a total acidity of 6.41 g/L. Moreover, gluconic acid was not detected in the three grape samples.

### Viable and Cultivable Microbial Community after Fungicide Applications

The culture media employed in this study were selected to make possible the quantification of species usually involved in the vinification process. Therefore, the GYP was employed for quantifying total yeasts, DBDM for *Brettanomyces/Dekkera*, MRS for LAB, and Mann for AAB and EB.

**FIGURE 1:**
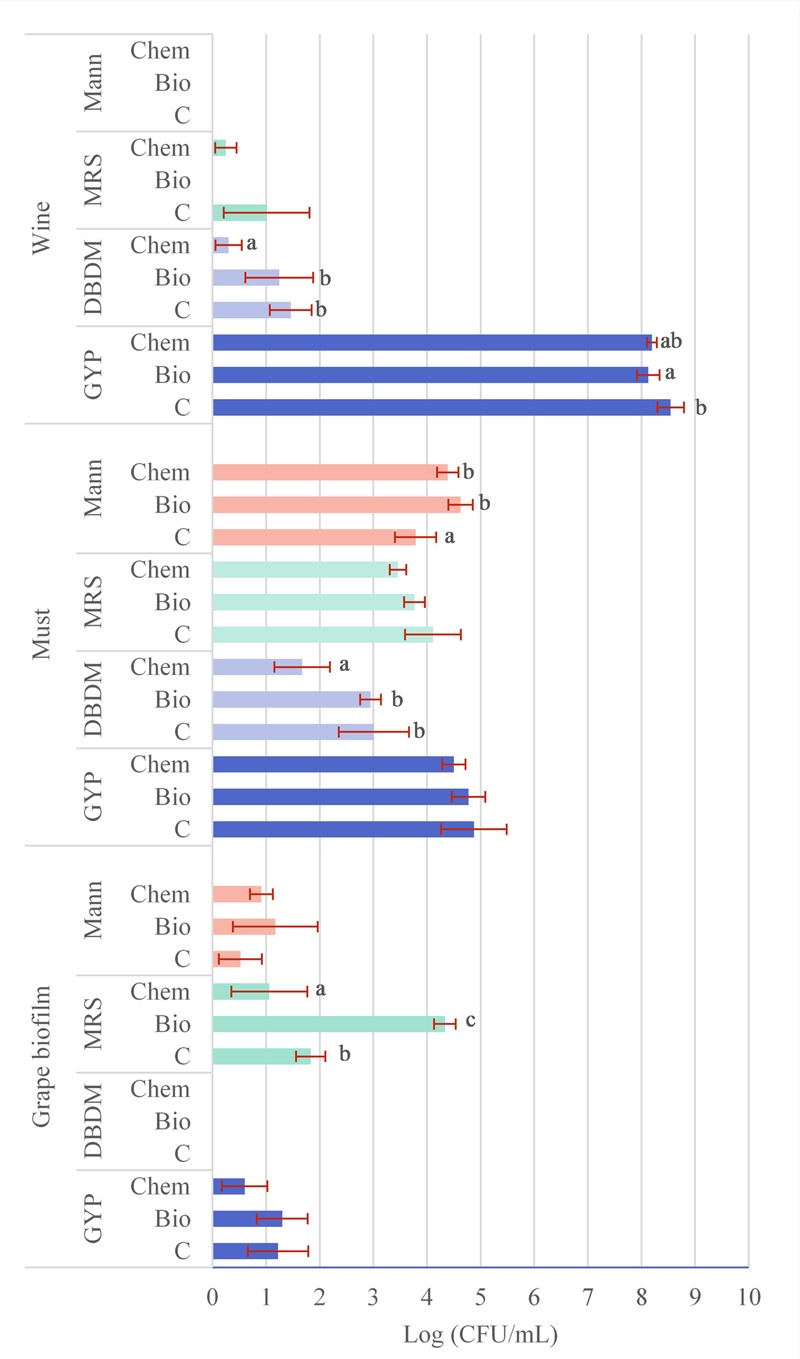
Average viable and cultivable (VC) microbial community in log units (CFU/mL) counted on plates of different samples (C: control, Bio: bio-fungicide, and Chem: chemical fungicide) at different stages (1: grape biofilm; 2: must, 3: wine) and from different culture media (GYP, DBDM, MRS, and Mann), the statistical analysis (different letters mean significant differences *p* < 0.05 between treatments) and error bars. Empty bars mean no colonies detected.

In **Figure 1**, the average VC community (log CFU/mL) found with the different culture media and at the different stages is shown. The VC microbial community of grape biofilm was determined with the culture media GYP, MRS, and Mann. The yeasts growing on GYP plates varied from 0.6 to 1.3 log units without statistical significance. Regarding the VC bacteria growing on MRS plates from grape biofilm, significant differences were established between control sample (1.8 log units) and both fungicide samples, having the bio-fungicide sample the highest VC community (4.3 log units) and the chemical product the lowest one (1.1 log units). The VC bacteria growing on Mann plates from grape biofilms was similar between samples (from 0.5 to 0.8 log units) so that significant differences were not determined.

Data linked to the average VC community (log CFU/mL) at must sampling are also shown in **Figure 1**. In this case, all culture media employed hold colonies growing. The VC yeasts of GYP plates were in the range of 4.5 and 4.9 log units and differences were not significant. Regarding the VC community growing on DBDM plates with the must from grapes treated with the chemical fungicide was significantly lower (1.7 log units) than the other two samples (3 log units). The VC bacteria on MRS plates was not significantly different between samples (from 4.1 to 3.5 log units) although on Mann plates with the control samples was significantly lower (3.8 log unit) than the determined in the other samples (4.4 and 4.6 log units).

Regarding the VC community (log CFU/mL) quantified at wine sampling (**Figure 1**) results were obtained from GYP, DBDM, and MRS plates. Yeasts growing on GYP plates ranged from 8.2 corresponding to wine from the bio-fungicide application, to 8.5 log units for control samples but without significant differences. Regarding the VC yeasts growing on DBDM plates, VC microbial community from chemical treatment was significant lower (0.3 log units) than in the other two samples (1.2–1.5 log units). Bacteria VC community on MRS plates was not significantly different between samples, being 1 log unit in control samples and 0.3 log unit in wines from chemical treated grapes and null for bio-fungicide treatment.

### Species Composing the Microbial Community

#### Grape Biofilm

**FIGURE 2:**
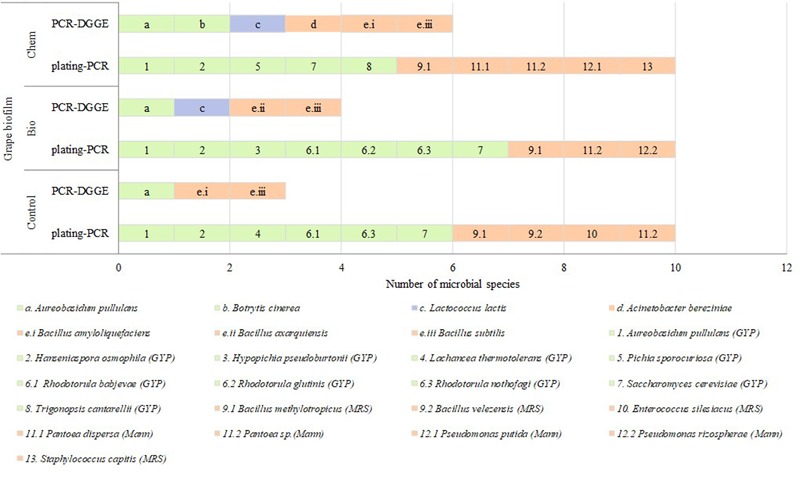
Microbial species detected on grape biofilm samples (C: control, Bio: bio-fungicide, and Chem: chemical fungicide) from GYP, MRS, and Mann plates referred to as letters and by PCR-DGGE referred to as numbers. Green slices identified eukaryotic species; purple slices were LAB; and orange slices EB.

In **Figure 2**, data about species found by culture dependent and independent methods in samples of grape biofilms are shown. On grape biofilm control sample, six yeasts – *Aureobasidium* (*A.*) *pullulans, Hanseniaspora* (*H.*) *osmophila, Lachancea* (*Lch*.) *thermotolerans, Rhodotorula* (*Rh*.) babjevae, *Rh. nothofagi* and *Saccharomyces (S.) cerevisiae-* and six EB -*Bacillus* (*B.*) *amyloliquefaciens, B. methylotropicus, B. subtilis, B. velezensis, Enterococcus* (*E*.) *silesiacus*, and *Pantoea sp.-* were identified. The species *B. amyloliquefaciens* and *B. subtilis* were detected with PCR-DGGE and ten species were isolated from GYP, MRS, and Mann plates.

After the application of the bio-fungicide, seven yeasts, one LAB and five EB were detected. Five of them were different regarding grape biofilm control sample: *Hypopichia (Hy.) pseudoburtonii, Rh. glutinis, Lactococcus (Lc.) lactis, B. axarquiensis, and Pseudomonas (Ps.) rizospherae-*. The species *Lc. lactis, B. axarquiensis*, and *B. subtilis* were detected with PCR- DGGE (**Supplementary Figure S1**) while ten species were isolated from GYP, MRS, and Mann plates.

The species of microorganisms after the application of the chemical fungicide were six yeasts, one LAB and eight EB. Eight of them were different to grape biofilm control sample -*Botrytis* (*Bo.*) *cinerea, Pichia* (*Pi.*) *sporocuriosa, Trigonopsis* (*Tr.*) *cantarellii, Lc. lactis, Acinetobacter* (*Ac.*) *bereziniae, Pantoea* (*P.*) *dispersa, Ps. putida, and Staphylococcus* (*St.*) *capitis-*. The species *Bo. cinerea, Lc. lactis, Ac. bereziniae, B. amyloliquefaciens*, and *B. subtilis* species were detected with PCR-DGGE (**Supplementary Figure S3**) while ten species were isolated from GYP, Mann, and MRS culture media.

#### Must

**FIGURE 3:**
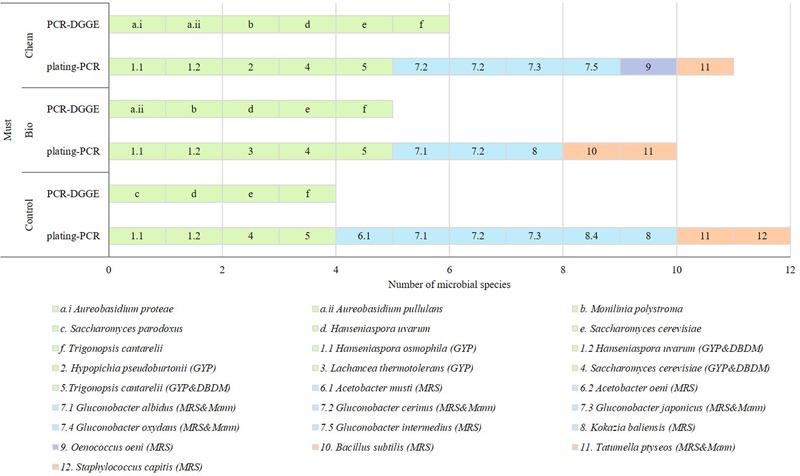
Microbial species detected in must samples (C: control, Bio: bio-fungicide, and Chem: chemical fungicide) from GYP, DBDM, MRS and Mann plates referred to as letters and by PCR-DGGE referred to as numbers. Green slices identified eukaryotic species; blue slices were AAB; purple slices were LAB; and orange slices EB.

In **Figure 3**, data about species found by culture dependent and independent methods in must samples are shown. In must control sample, five yeasts -*H. osmophila, H. uvarum, S. cerevisiae, S. paradoxus* and *Tr. cantarellii-*, six AAB -*Acetobacter* (*Ace.*) *musti, Gluconobacter* (*G.*) *albidus, G. cerinus, G. japonicus, G. oxydans* and *Kokazia* (*K.*) *baliensis-* and two EB –*St. capitis* and *Tatumella* (*Ta*.) *ptyseos*- were identified. In general, the yeast species detected with PCR-DGGE were also isolated from GYP and DBDM plates while bacteria were isolated from MRS and Mann plates.

The species of musts proceeding from grapes treated with the bio-fungicide were seven yeasts, three AAB and two EB and among them four species were different regarding must control sample -*A. pullulans, Lch. thermotolerans, Monilinia* (Mo.) *polystroma*, and *B. subtilis-*. Most of the yeast species were identified with PCR-DGGE and isolated from GYP and DBDM plates. The bacteria were isolated from MRS and Mann plates.

Musts of grapes treated with the chemical fungicide contained eight yeasts, four AAB, one LAB, and one EB; seven species were different to those of must control sample -*A. proteae, A. pullulans, Hy. pseudoburtonii, Mo. polystroma, Ace. oeni, Gl. Intermedius*, and *Oenococcus* (*O*.) *oeni-*. Some of the detected yeasts proceeded from DGGE gels and they were isolated from GYP and DBDM culture media while all the bacteria were isolated from MRS and Mann plates.

#### Wine

**FIGURE 4:**
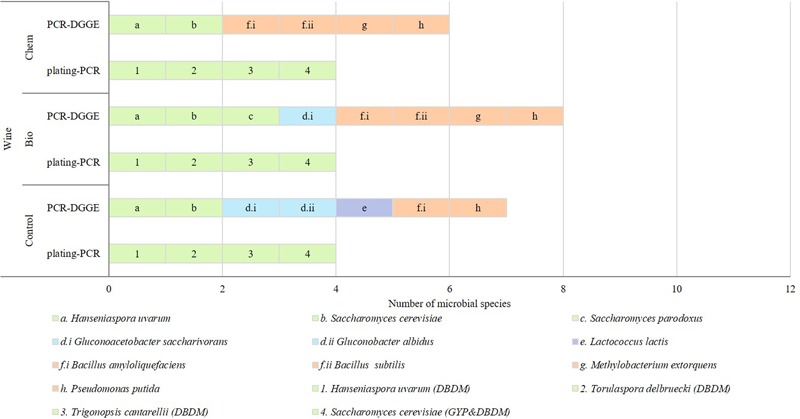
Microbial species detected in wine samples (C: control, Bio: bio-fungicide, and Chem: chemical fungicide) from GYP and DBDM plates referred to as letters and by PCR-DGGE referred to as numbers. Green slices identified eukaryotic species; blue slices were AAB; purple slices were LAB; and orange slices EB.

In **Figure 4**, species found by culture dependent and independent methods in wine samples are shown. Nine species were identified in wine control sample, being four yeasts –*H. uvarum, S. cerevisiae, S. paradoxus*, and *Tr. cantarellii-*, two AAB –*Gluconoacetobacter* (*Ga.*) *saccharivorans* and *G. albidus*-, one LAB -*Lc. lactis-* and two EB -*Methylobacterium* (*Me.*) *extorquens* and *Ps. putida-*. All the species were found with PCR-DGGE (**Supplementary Figures S1–S3**) and two yeasts were isolated from GYP and DBDM plates.

The wine proceeding from grapes treated with the bio-fungicide had five yeast, one LAB and four EB species, seven out of them were common with the found in wine control samples while three were different -*Torulaspora* (*To*.) *delbrueckii, B. amyloliquefaciens*, and *B. subtilis-*. All the species were found with PCR-DGGE and two yeasts were isolated from GYP and DBDM plates.

The wine from grapes treated with the chemical fungicide contained three yeast, one LAB and four EB species, six species appeared either in wine control samples while *B. subtilis* and *Me. extorquens* were different. The bacteria and two of the yeast were detected with PCR-DGGE and four yeasts were also isolated from GYP and DBDM plates.

### Microbial Alpha Diversity of the Samples

The average diversity indexes assessed with the data of species identified in each replicate for describing the alpha diversity of the samples are shown in **Table 1**. Significant differences were not established between the diversity indexes of grape biofilm samples. The Margalef index ranged from 2.32 to 3.22, the Simpson index from 0.77 to 0.86, the Berger–Parker index from 0.14 to 0.23, the Shannon–Wiener index (*H*) from 1.50 to 2.00 and the Chao-1 from 13 to 33.

**Table 1:** Microbial alpha diversity indexes (C: control, Bio: bio-fungicide, and Chem: chemical fungicide) assessed for the samples (grape biofilm, must, and wine), and statistical analysis at the same stage.

Samples		Species richness	Structure of community			
		Margalef	Simpson	Berger–Parker	*H*	Chao-1
Grape biofilm:	C	2.48	0.80	0.20	1.60	15
	Bio	3.05	0.83	0.17	1.87	32
	Chem	3.22	0.86	0.14	2.00	33
Must:	C	3.08	0.85	0.15	1.94	29
	Bio	3.08	0.85	0.15	1.94	29
	Chem	3.07	0.85	0.15	1.92	29
Wine	C	3.37b	0.88b	0.13a	2.08b	36ab
	Bio	3.51b	0.89b	0.12a	2.14b	41b
	Chem	2.71a	0.82a	0.18b	1.73a	20a

*Different letters mean significant differences (*p* < 0.05) between the diversity indexes of samples at the same stage.*

In the diversity indexes of must samples, significant differences were not observed, being results the same with the exception of the Margalef index from 3.07 to 3.08 and the Shannon–Wiener index (*H*) from 1.93 to 1.94.

Diversity indexes at wine stage were in some cases significantly different between treatments. The wine control sample had similar indexes regarding the species richness and the structure of microbial community of the wine from the bio-fungicide application. The Margalef index, the Simpson index, the Shannon–Wiener index (*H*), and the Chao-1 were significantly lower in wine from grapes treated with the chemical fungicide than the determined for the other samples. The Berger–Parker index of wine from grapes treated with the chemical fungicide was significantly higher than the assessed for the other samples.

**FIGURE 5:**
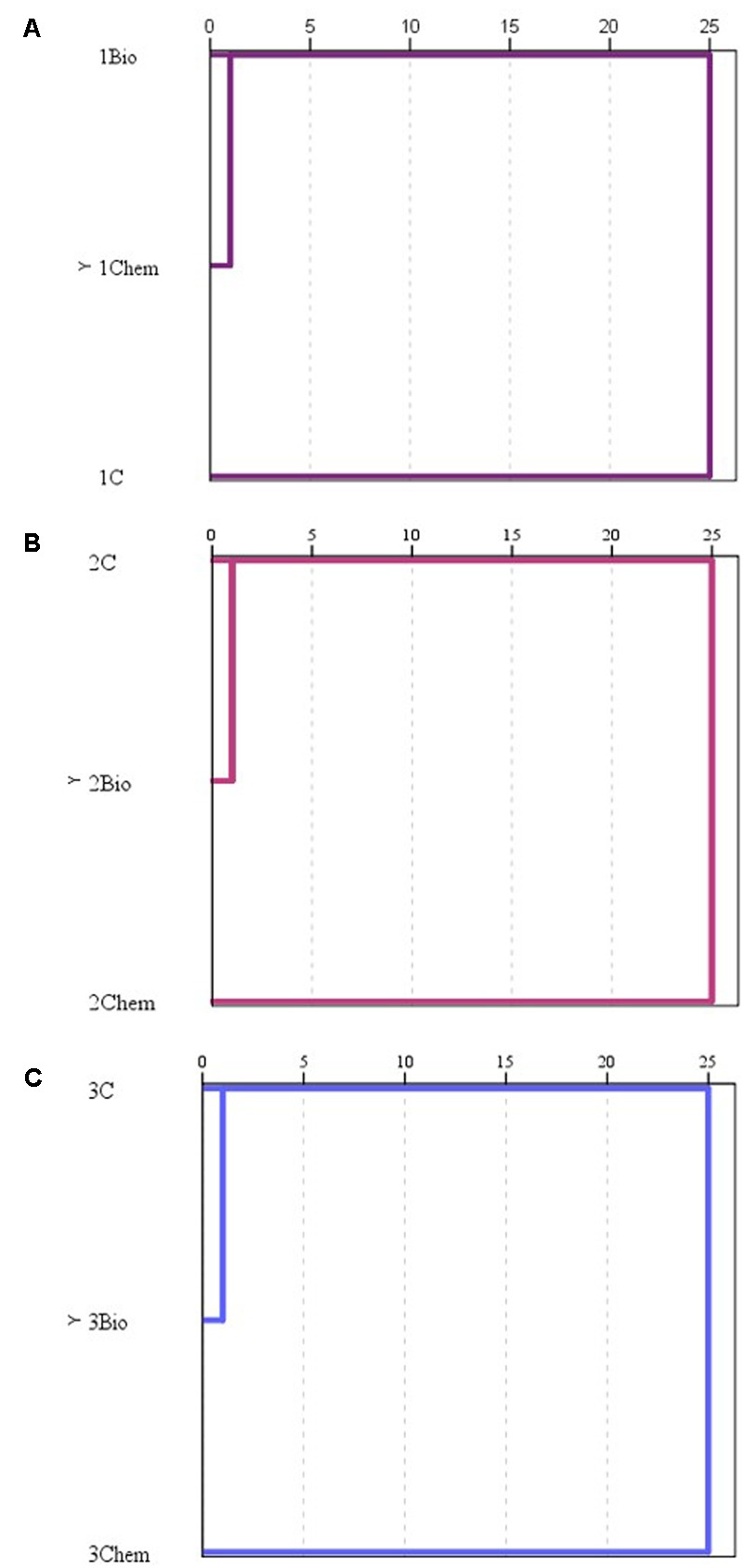
Hierarchical cluster of the diversity indexes of the samples (C: control, Bio: bio-fungicide, and Chem: chemical fungicide) assessed for the grape biofilm **(A)**, for the must **(B)**, and for the wine stage **(C)**.

Hierarchical clusters built with the diversity indexes of samples at each stage are shown in **Figure 5**. With this statistical analysis, it was observed that at grape biofilm stage the samples from fungicide treatments were clustered together. At the other two sampling moments, control samples were clustered together with samples proceeding from grapes treated with the biological fungicide whereas samples from grapes treated with the chemical fungicide stayed separately.

### Alcoholic Fermentation

The kinetics of spontaneous AF of all samples were studied by the determination of the daily density and they lasted 13 days. No differences were observed in samples of wines proceeding from grapes treated with fungicides application compared to the control (data no shown).

## Discussion

The current research was aimed to determine the impacts of the application of a bio-fungicide on the microbial community of the winemaking.

The bacterium *Bacillus subtilis* is one of the most interesting biological control agent against *B. cinerea*, it might be a tool for developing a more eco-friendly and sustainable viticulture. In effect, it has been described as a natural source of bioactive molecules against several mould diseases of the plants. Furthermore, it has the ability to generate spores, what makes this genus even more adequate to be applied in open and harsh environments having a long shelf-life being even applied along with chemical fungicides (Ongena and Jacques, 2008; Pertot et al., 2017; Reiss and Jørgensen, 2017). Precisely, Reiss and Jørgensen (2017) have recently reported the activity of the strain QST713 of the species *Bacillus subtilis* against the fungi *Puccinia striiformis* in preventive and curative way. This strain QST713 is the most important agent in abundance in the tested bio-fungicide (Serenade^®^ Max, Bayer Crop Bioscience S.L).

Regarding fenhexamid application some authors have described slow AFs probably because an impact on *S. cerevisiae* (Bizaj et al., 2014). However, other authors have not observed this effect (Cabras et al., 2004) and so far, no results have been published on the impact of fungicides on malolactic fermentation or on *O. oeni.*

The current study deals with the effect of *B. subtilis* strain QST713 on the microbiota at three stages of the vinification, grape biofilm, musts and wines. Results were compared to the impact of a traditionally employed chemical fungicide and to a non-treated control sample. Actually, no published research has dealt with this issue despite it could cause a significant impact on the winemaking if the ecological balance of the microbial populations were eventually affected by the biological control bacterium.

For this purpose, culture-dependent and independent techniques were employed. Culture dependent techniques allowed the detection of the microorganisms VC in culture medium under specific conditions. The PCR-DGGE approach allowed the detection of DNA proceeding from both alive and dead cells. Therefore, both techniques have quite important limitations to take into consideration. Moreover, the two methods of analysis do not generally give the same results for the same sample. The detection limits of PCR-DGGE technique have been usually considered higher than culture-dependent techniques even more with mixed populations (Bester et al., 2010). Nevertheless, in previous studies it was determined that, even with counts lower than 10^1^ CFU/mL, this culture-independent method provided interesting results (González-Arenzana et al., 2017b). Consequently, the combination of both approaches could reach a wide insight in the microbial community.

The initial oenological conditions of grapes were similar between samples and significant differences were not found after statistical analysis of analytical data. Furthermore, grape samples had not evidence of being infected by *Botrytis cinerea*, so that the health grape state was adequate.

### Impact of Fungicides on Grape Biofilm

In general, focusing on VC microbial community of grape biofilms, small populations of yeasts and bacteria were observed, accordingly to the described by Renouf et al. (2005). Apparently, the employment of both fungicides did not exert a dramatic change either in VC yeasts or in the yeast genera detected by culture-dependent and independent techniques. For example, the species *S. cerevisiae* was found in all samples although its detection in the grapevine environment with so small yeast population is considered really difficult (Renouf et al., 2005). Moreover, the yeast *A. pullulans* that is said to be a natural antagonist of the gray mold was present in all samples (Pertot et al., 2017). Additionally, the yeast *H. osmophila* was also found in all the samples being easily detectable at early stages of the vinification (Garijo et al., 2011). The combination of *Saccharomyces* and non-*Saccharomyces* genera in grape biofilm control sample was similar in grapes with the bio-fungicide. Nevertheless, some differences were observed in comparison to the grape biofilms with the chemical fungicide. For instance, the gray mold or *B. cinerea* was detected by culture-independent methods, not being found either in control sample, or in the grapes with the bio-fungicide.

Regarding the VC bacteria community after the application of the bio-fungicide, it was significantly higher than for control and chemical fungicide samples what would be due to the ability of *Bacillus* for growing on MRS plates (González-Arenzana et al., 2017a). Thus, viability of *Bacillus* cells applied to the grapevine was corroborated in the current study being found in great percentage in samples from bio-fungicide application and being also detected in the other two samples. Precisely, *Bacillus subtilis* is an EB gram-positive ubiquitous bacterium in the nature, similarly to *Pantoea* genus that was also found in some samples of this study. Furthermore, *Pseudomonas* genus was identified in all the samples after the application of both fungicides while in control sample it was not found. This genus is also an EB that has been described for playing an important role in both the grape biofilm formation and in the biological control of some spoilage microorganisms linked to grape surface (Renouf et al., 2005). Overall, genera of bacteria usually involved in winemaking such as AAB and LAB were not abundant on the grape surface. A case in point are the AAB usually linked to grape surface diseases, such as the gray mold (Barata et al., 2012) that were not detected at this first sampling probably because of the good health status of grapes. The only LAB detected was the genus *Lactococcus* recently described also in the grape surface (González-Arenzana et al., 2017a).

Apart from genera and species identified on grape surface, the microbial alpha diversity was numerically assessed. The microbial community of the grape biofilms was not significantly modified after the application of both fungicides. This result is contrary to others reporting significant changes in yeast and bacteria community of grape surfaces after applying copper based fungicides (Martins et al., 2012, 2014). Authors such as Cordero-Bueso et al. (2011) have reported higher diversity of the microbial communities of grapes after the organic management of grapevine, but in the current study this result was not clearly established. In fact, statistical differences in the richness of species or in the structure of the microbial community of the three different samples were determined neither after the application of the bio-fungicide, nor after the application of the chemical fungicide. Only in the hierarchical cluster, both fungicide treatments were clustered in the same branch.

### Impact of Fungicides on Must Microbiota

Must microbial community is a mixture of microorganisms from grape biofilms and of microorganisms from winery facilities. Focusing on must from crushed, destemmed and sulphited grapes, it was observed that VC community experienced two significant changes linked to the fungicide applications. On one hand, yeast community on DBDM plates, proceeding from chemical fungicide, were lower than the one counted on plates from control and bio-fungicide treated samples. This culture media did not provide information about *Dekkera* and *Brettanomyces* genera despite being prescribed for their detection (Rodrigues et al., 2001). On another point, the genera detected by DBDM plates were mostly found by GYP, so significant differences found by DBDM were not representative of the total yeast community. Yeasts on all the GYP plates corresponded with the commonly found in the literature about early stages of spontaneous AF, around 5 log units (Gutiérrez et al., 2001). Yeast genera identified in must from grapes treated with both fungicides were qualitatively similar. The genera *Hanseniaspora, Saccharomyces*, and *Trigonopsis* were found in all musts. In must sample control, *S. paradoxus* was detected along with *S. cerevisiae* what might deteriorate the organoleptically features of wines if both acted in the AF, according to the described by Alonso-del-Real et al. (2017). The genera *Aureobasidium* was present in musts after the fungicide treatments what could enhance the effectivity of the treatments, because it is thought to be a natural antagonist of some moulds affecting grapevine health state. Contrary to this, *Mo. polystroma*, which is a cherry pathogen (Poniatowska et al., 2016), was detected only after both fungicide application. In this case, these type of contradictory results made very difficult to establish some clear impact of agronomic treatments on microbial communities of musts.

Most of the bacteria found at must stage grew on MRS and Mann culture media, but AAB were mainly found in Mann with significantly lower populations in control sample. AAB presence in musts could proceed from the winery environment, because in grape biofilms were not detected. Definitely their detection at this stage is considered negative for wine organoleptic characteristics (Guillamón and Mas, 2011). The number of AAB species in must from grapes that were biologically treated were lower than in the other samples what might mean a positive impact of the bio-fungicide. In a similar way, *Oenococcus* was the only LAB in must from grapes treated with the chemical fungicide but it also could come from the winery environment because it was not found in the previous sampling stage. The number of EB species was lower than the observed in grape biofilms being only *Ta. ptyseos* detected in all the must samples. It is a rare food borne pathogen that causes some human infections (Mardaneh et al., 2014) and its origin must be located on the winery. This is the first time that this genus has been reported in must samples being traditionally found in the coffee fermentation (Silva et al., 2008). The presence of *B. subtilis* only in must from grapes treated with the bio-fungicide could be indicating that this genus was able to resist the operations performed in the vinifications process even the sulphiting. These results were even more interesting because these bacteria were isolated in a VC form.

Statistically, the microbial alpha diversity of must samples was equal in most of the assessed indexes, showing only slight differences between samples. In spite of being clearly similar regarding indexes of microbial alpha diversity, the hierarchical cluster of must samples clustered together the control samples with the must proceeding from the bio-fungicide treatments, staying the chemical fungicide must apart. This could indicate that the chemical fungicide applied to grapes might exert some kind of impact on microbial community at must stage that made this sample slightly different to the other two samples.

### Impact of Fungicides on Wine Microbiota

Each must successfully underwent through spontaneous AF regardless the type treatment applied to grapes. Regarding microbial community when AF was completed, great yeast populations on GYP plates were observed in all samples. At this point of winemaking, the yeast community, especially the *S. cerevisiae* population, was so high that the other microorganisms stayed in a secondary place. Again, likewise musts, yeasts on DBDM plates of wine from grapes treated with the chemical fungicide were significantly minor than yeasts of the other samples but their identification provided interesting information because genera different to *Saccharomyces* were detected. For instance, *H. uvarum* was again found by culture dependent and independent methods in all the wine samples. Furthermore, wine proceeding from grapes treated with the bio-fungicide were qualitatively more diverse than the other because *To. delbrueckii* and *Tr. cantarelli* species were also found. *To. delbrueckii* had not been detected in previous stages, so its presence in wines after the AF could be indicating an important population during this harsh stage probably due to its resistance to factors such as sulfur dioxide, as it has been demonstrated in recent studies (González-Arenzana et al., 2017a). Cordero-Bueso et al. (2011) reported this increase in the qualitative diversity on grape biofilm after organic agronomic practices.

In relation to bacteria community growing on MRS and Mann plates, important differences between fungicides and control samples were not noticed, but culture-independent methods provided some important results about bacteria community. An obvious example was the LAB *Lc. lactis* that was detected in all samples with PCR-DGGE. This result was in accordance to the previously described in wines from this same region in which several LAB species, including *Lc. lactis*, were found after the AF in a non-cultivable form (González-Arenzana et al., 2017b). The EB identified in wines were also in a non-cultivable form, thus, for instance, *Me. extorquens* and *Ps. putida* were detected in all samples and *Bacillus* was found in samples proceeding from both fungicide treatments. The presence of *Methylobacterium* has been recently reported by Portillo et al. (2016) in the grape surface of different varieties; and *Ps. putida*, present in grapevine soil, is very important because its capacity of resisting different metal contamination (Chong et al., 2016). Furthermore, the identification of *Bacillus subtilis* applied from grapevine until the AF depletion should be taken into consideration in future works.

At this sampling stage, differences in the microbial alpha diversity of wines were established in samples proceeding from grapes treated with the chemical fungicide. This sample was significantly different from the other two, having lower richness of species (Margalef index), lower diversity (Shannon–Wiener index, H) and lower number of odd species (Chao-1). This would have made these samples more sensitive to external threats than other samples with higher diversity of microorganisms. In contrast, the possibility of finding two randomly selected individuals belonging to the same species was lower in this sample and the equity assessed with the Berger-Parker index was also lower. This means that chemical fungicide samples were balanced in terms of structure of the microbial community in spite of having lower richness of species (Provost and Pedneault, 2016). The hierarchical cluster was approximately the same than the described for musts although the indexes of the alpha diversity were much more differenced at wine stage. Thus, clustering was very useful to corroborate the statistical analysis.

## Conclusion

On balance, the application of the bio-fungicide in the grapevine caused an increase in viable microbial community growing on the MRS culture media in grape surface; in contrast, it did not affect significantly the microbial alpha diversity of the grape biofilm. Some of the *Bacillus* applied with the bio-fungicide were detected at must stage, and the microbial alpha diversity of this sample was more similar to the determined for control than the determined for must from grapes treated with the chemical fungicide. Despite these slight effects, the spontaneous AF was developed without problems in all the samples. Nevertheless, the microbial alpha diversity was very different for the wine from grapes treated with the chemical fungicide whereas control and wine from grapes treated with *Bacillus* were quite similar with higher species richness and quite similar structure of the microbial community based on a high diversity but also a high dominance of some species.

To sum up, under the conditions of this experiment (no contamination by *B. cinerea*) and with the analysis protocols used, the results showed that the biofungicide had no impact on alpha microbial diversity until the end of fermentation. If confirmed in other environmental and analytical conditions, this biofungicide could be applied to the vine as a biological control of the grey grape rot.

## Author Contributions

RE-V has been in charge of the acquisition, analysis, or interpretation of data for the work. RL and PS have made substantial contributions to the conception or design of the work. LG-A, AG, RL, and IL-A have revised the work critically for important intellectual content. All the authors have provided the final approval of the version to be published and therefore, are in agreement to be accountable for all aspects of the work in ensuring that questions related to the accuracy or integrity of any part of the work are appropriately investigated and resolved.

## Conflict of Interest Statement

The authors declare that the research was conducted in the absence of any commercial or financial relationships that could be construed as a potential conflict of interest.

## Supplementary Material

FIGURE S1 Some of the WLAB1-2 amplicons and species detected by DGGE in control replicates (C1, C2, C3, and C4), in samples form biofungicide treatment (B1, B2, B3, and B4) and in samples from chemical fungicide treatment (Ch1, Ch2, Ch3, and Ch4).

FIGURE S2 Some of the WBAC 1-2 amplicons species detected by DGGE in control replicates (C1, C2, C3, and C4), in samples form biofungicide treatment (B1, B2, B3, and B4) and in samples from chemical fungicide treatment (Ch1, Ch2, Ch3, and Ch4).

FIGURE S3 Some of the NL1-LS2 primers amplicons species detected by DGGE in control replicates (C1, C2, C3, and C4), in samples form biofungicide treatment (B1, B2, B3, and B4) and in samples from chemical fungicide treatment (Ch1, Ch2, Ch3, and Ch4).
