# Supplementary figures and images for: Impact of Chemical and Biological Fungicides Applied to Grapevine on Grape Biofilm, Must, and Wine Microbial Diversity
Source: Front Microbiol. 2018 Feb 2;9:59. doi: 10.3389/fmicb.2018.00059 (PMC5808214; doi:10.3389/fmicb.2018.00059)

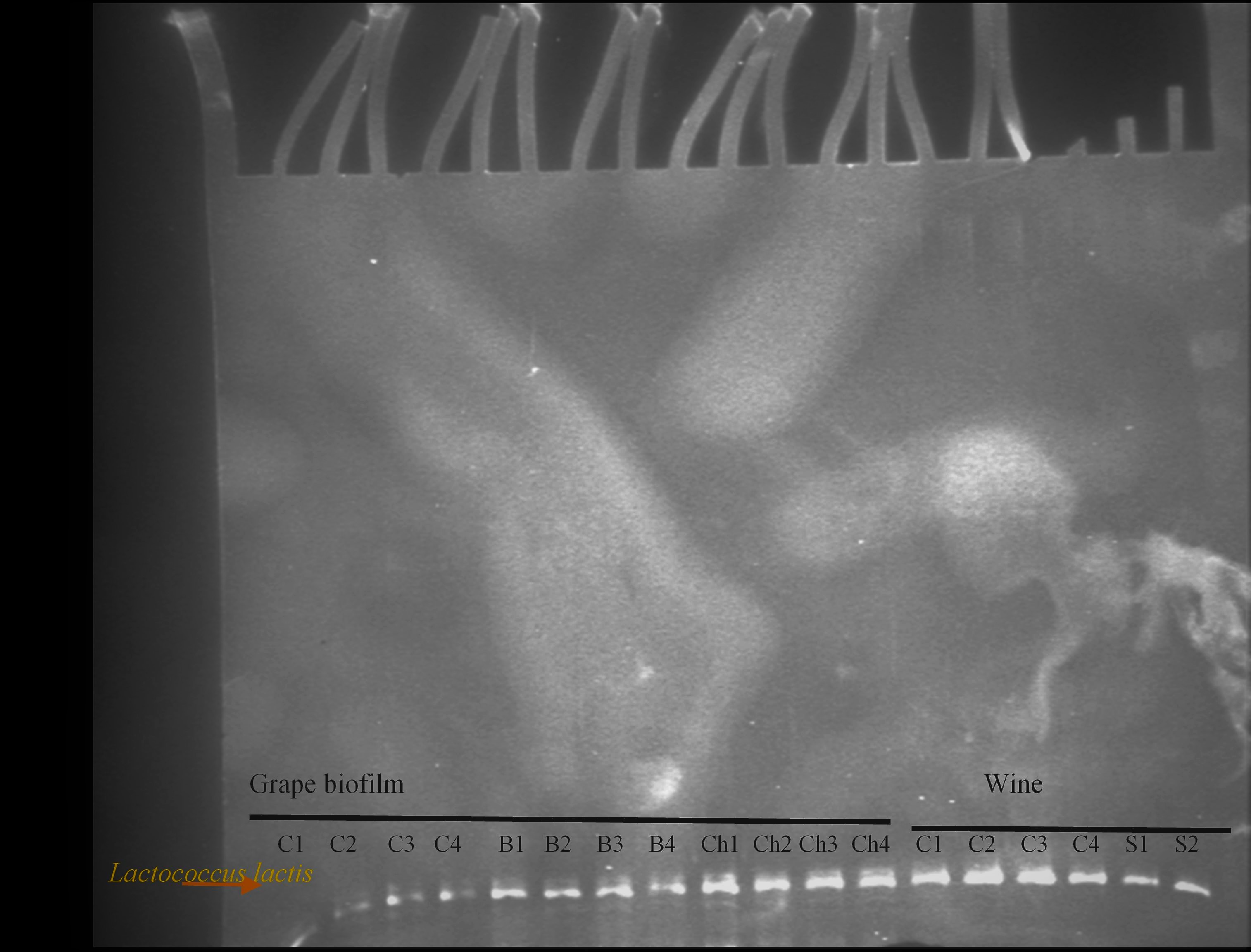

Supplement: FIGURE S1 — Some of the WLAB1-2 amplicons and species detected by DGGE in control replicates (C1, C2, C3, and C4), in samples form biofungicide treatment (B1, B2, B3, and B4) and in samples from chemical fungicide treatment (Ch1, Ch2, Ch3, and Ch4). [file Image_1.JPEG]

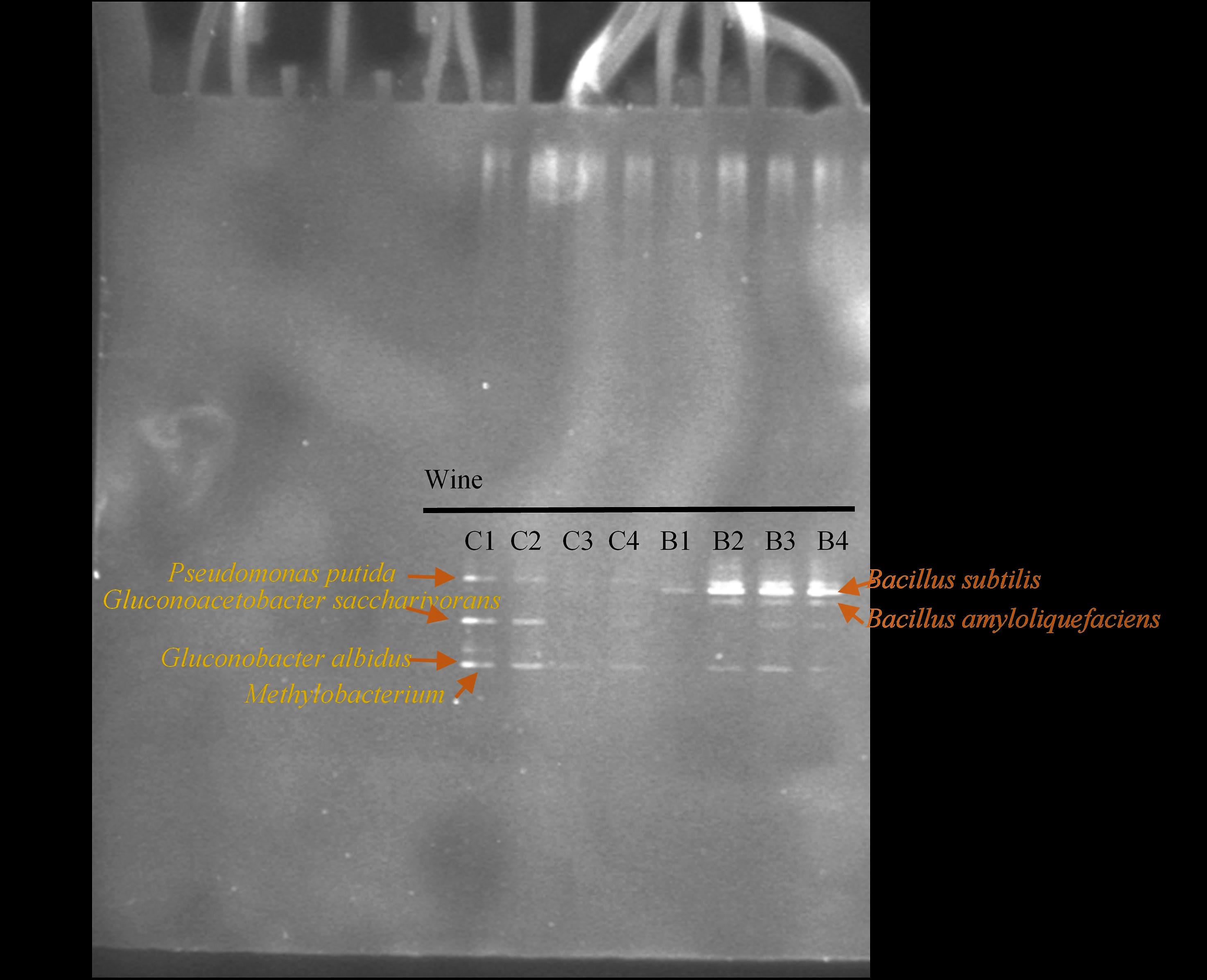

Supplement: FIGURE S2 — Some of the WBAC 1-2 amplicons species detected by DGGE in control replicates (C1, C2, C3, and C4), in samples form biofungicide treatment (B1, B2, B3, and B4) and in samples from chemical fungicide treatment (Ch1, Ch2, Ch3, and Ch4). [file Image_2.JPEG]

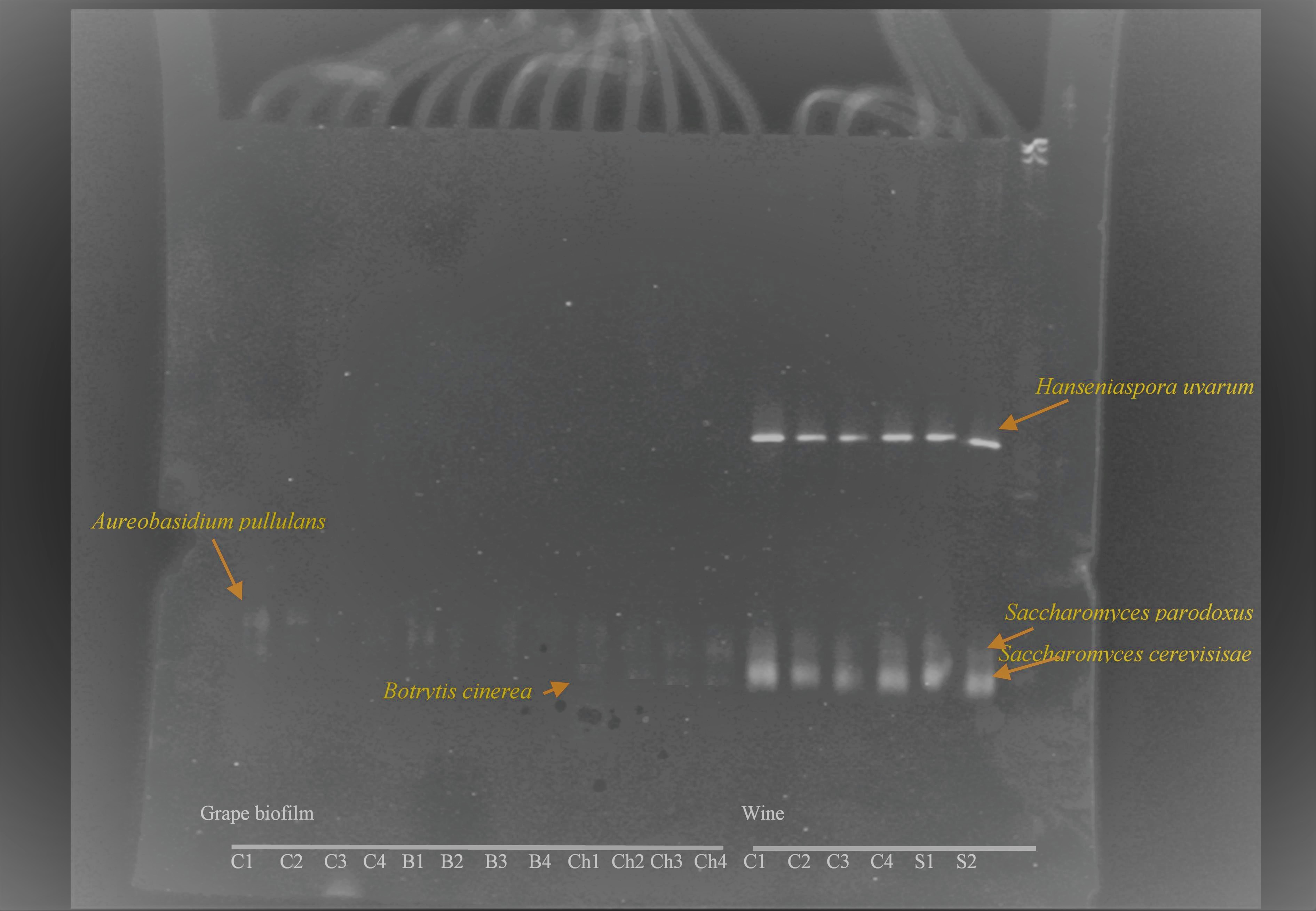

Supplement: FIGURE S3 — Some of the NL1-LS2 primers amplicons species detected by DGGE in control replicates (C1, C2, C3, and C4), in samples form biofungicide treatment (B1, B2, B3, and B4) and in samples from chemical fungicide treatment (Ch1, Ch2, Ch3, and Ch4). [file Image_3.JPEG]
